# Oral microbiome changes associated with the menstrual cycle in healthy young adult females

**DOI:** 10.3389/fcimb.2023.1119602

**Published:** 2023-03-31

**Authors:** Ayaka Yamazaki, Kohei Ogura, Kana Minami, Kazuhiro Ogai, Tomomi Horiguchi, Shigefumi Okamoto, Kanae Mukai

**Affiliations:** ^1^ Division of Transdisciplinary Sciences, Graduate School of Frontier Science Initiative, Kanazawa University, Kanazawa, Japan; ^2^ Advanced Health Care Science Research Unit, Innovative Integrated Bio-Research Core, Institute for Frontier Science Initiative, Kanazawa University, Kanazawa, Japan; ^3^ Department of Health Development Nursing, Institute of Medical, Pharmaceutical and Health Sciences, Kanazawa University, Kanazawa, Japan; ^4^ AI Hospital/Macro Signal Dynamics Research and Development Center, Institute of Medical, Pharmaceutical, and Health Sciences, Kanazawa University, Kanazawa, Japan; ^5^ Department of Clinical Nursing, Faculty of Health Sciences, Institute of Medical, Pharmaceutical and Health Sciences, Kanazawa University, Kanazawa, Japan; ^6^ Department of Clinical Laboratory Sciences, Faculty of Health Sciences, Institute of Medical, Pharmaceutical, and Health Sciences, Kanazawa University, Kanazawa, Japan

**Keywords:** menstrual cycle, oral microbiome, saliva, *Prevotella*, *Streptococcus*

## Abstract

The relationship between the menstrual cycle and the oral microbiome has not been clarified. The purpose of this study was to assess potential changes in the oral microbiome of healthy young adults using 16S rRNA-based sequencing. Eleven females (aged 23–36 years) with stable menstrual cycles and without any oral problems were recruited. Saliva samples were collected before brushing every morning during the menstrual period. Based on basal body temperatures, menstrual cycles were divided into four phases, namely the menstrual, follicular, early luteal, and late luteal phases. Our results showed that the follicular phase had a significantly higher abundance ratio of the *Streptococcus* genus than the early and late luteal phases, whereas the abundance ratios of the *Prevotella* 7 and *Prevotella* 6 genera were significantly lower in the follicular phase than those in the early and late luteal phases and that in the early luteal phase, respectively. Alpha diversity by the Simpson index was significantly lower in the follicular phase than that in the early luteal phase, and beta diversity showed significant differences among the four phases. Using the relative abundance data and copy numbers of the 16S rRNA genes in the samples, the bacterial amounts in the four phases were compared, and we observed that the follicular phase had significantly lower amounts of the *Prevotella 7* and *Prevotella* 6 genera than the menstrual and early luteal phase, respectively. These results indicate reciprocal changes with the *Streptococcus* genus and *Prevotella* genera, particularly in the follicular phase. In the present study, we showed that the oral microbiome profiles are affected by the menstrual cycles of healthy young adult females.

## 1 Introduction

A menstrual cycle begins with the onset of menstrual flow. The menstrual phase generally lasts for 4–6 days with the shedding of the thickened endometrium, which is also known as menstrual bleeding. The follicular or proliferative phase continues until ovulation. And the luteal, or secretory phase begins at ovulation and continues until the onset of the next menstrual flow (Muizzuddin et al., 2006).

What has recently come to light is that the fluctuation of two hormones, estradiol and progesterone, plays a crucial role in neurological and psychological development and function, which impacts brain function, cognition, emotional status, sensory processing, and appetite (Farage et al., 2008). The menstrual cycle comes periodically with these physiological changes from menarche until menopause, except during pregnancy and childbearing. Because changes in these female hormones induce a variety of effects on the female’s physical and mental health, it is important to clarify the relationship between physical and psychological changes associated with the menstrual cycle and the mechanisms of their effects in order to maintain and promote individual health and wellbeing.

The oral cavity, which is the gateway to the gastrointestinal tract, communicates with the outside environment and contains as many as 5–10 billion bacteria, comprising a unique commensal microbiome (Dewhirst et al., 2010). In fact, the oral cavity harbors the second most abundant microbiota next to the gastrointestinal tract. According to the expanded Human Oral Microbiome Database (eHOMD) (updated in November 2017) (Chen et al., 2010), approximately 770 bacterial genera have been identified as oral commensals. It is well known that altered oral microflora has been observed in several diseases such as diabetes, bacteremia, endocarditis, cancer, autoimmune disease, and preterm births. Therefore, it becomes crucial to understand the fluctuation of the oral microbial diversity under diseased/perturbed conditions (Verma et al., 2018).

The relationship between pregnancy and oral microbiome has been previously summarized in literature; oral microbiome composition can contribute to pregnancy complications (Saadaoui et al., 2021). The pregnant body is affected by a series of hormonal, metabolic, and immunological changes, resulting in the divergence of the oral microbiome composition (Lain and Catalano, 2007; Wang et al., 2016). Multiple studies have examined microbiome differences between pregnant and nonpregnant females (Basavaraju et al., 2012; Borgo et al., 2014; Fujiwara et al., 2017). One of these studies showed that the total number of viable oral microbes was significantly increased in all stages of pregnancy compared to nonpregnant females (Fujiwara et al., 2017). Moreover, it has been found that the growth and proliferation of multiple bacterial taxa, such as *Lactobacillus*, *Bifidobacterium*, *Streptococcus*, and *Escherichia* genera, were altered during pregnancy (Pelzer et al., 2012).

While there are many studies on pregnancy and oral contraceptives, few studies have examined the effects of menstruation on the oral microbiome. Balan et al. reported that as many as 43% of females experience some oral symptoms during their menstrual cycle (Balan et al., 2012). Other groups showed an increase in inflammatory cytokines, such as IL-1β and TNF-α, in the saliva before menstruation (Baser et al., 2009; Khosravisamani et al., 2014). Furthermore, Machtei et al. found that the gingival index was significantly higher during ovulation and premenstruation than during menstruation, even though the mean plaque indices were similar at all time points (Machtei et al., 2004). These reports indicate that the oral environment changes in accordance with the menstrual cycle (Gao et al., 2018). To date, the relationship between oral microbiome and menstrual cycles remains uncertain. Fischer et al. concluded that there was no specific pattern in bacterial changes during the menstrual cycle (Fischer et al., 2008), while Calil et al. reported that anaerobic bacteria counts remained unchanged (Calil et al., 2008). On the contrary, Kawamoto et al. showed that *Prevotella intermedia* (*P. intermedia*) and *Porphyromonas gingivalis* (*P. gingivalis*) were significantly higher during the ovulatory phase compared to the follicular phase (Kawamoto et al., 2010; Kawamoto et al., 2012).

While these reports on menstrual cycles used culture-based methods, Bostanci et al. have recently conducted a metagenomics-based study (Bostanci et al., 2021). Saliva samples were collected from 103 females of reproductive age during the menstrual, follicular, and luteal phases of the cycle (n = 309), and the abundances of oral bacteria were analyzed by 16S rRNA V3–V4 sequencing. No significant differences in α-diversity or clustering were observed among the three phases. Nonetheless, the authors found that the richness of *Campylobacter*, *Hemophilus*, *Prevotella*, and *Oribacterium* genera varied throughout the cycle, especially during the luteal phases.

The relationship between menstrual cycle and oral microbiome has not been clarified. Therefore, the purpose of this study was to clarify the changes in the oral microbiome during the menstrual cycle in 11 healthy young adult females. One of the pitfalls of oral microbe studies involves the precise timing of sample collection because oral samples are affected by brushing and ingesting drinks/foods. In order to unify collecting conditions, saliva samples were collected daily for approximately one month from the start of menstruation, after waking up and before brushing.

## 2 Materials and methods

### 2.1 Criteria of subject selection

Female researchers recruited 11 (Subjects #F1-F11) females (aged 23–36 years) with stable menstrual cycles, who had not taken any oral contraceptives or antibiotics within one month prior to the beginning of this study (**Table S1**). Five (Subjects #M1–M5) males (aged 22–32 years), who had not taken any antibiotics within one month prior to the beginning of this study, were recruited as controls. The participants were staffs, undergraduate students, and graduate students.

### 2.2 Saliva collection

Every morning, two milliliters of saliva were collected from each participant into a drool collection kit before brushing teeth. The saliva samples were transferred into storage tubes and stored in a freezer. For DNA extraction, 100 μL of whole saliva was diluted with 400 μL of phosphate buffered saline to reduce viscosity, and the diluted saliva was transferred to a Pathogen Lysis Tube S (QIAGEN N.V.) and mechanically disrupted for 10 min using a Disruptor Genie (Scientific Industries). The resultant solution was subsequently processed using the QIAamp UCP Pathogen Mini Kit (QIAGEN N.V.) according to the manufacturer’s instruction. Double stranded DNA concentrations were measured using the Qubit HS Assay Kit (ThermoFisher SCIENTIFIC). Additionally, the collected saliva was thawed and centrifuged at 1500 g for 15 min, and the supernatants were utilized in an ELISA testing below.

### 2.3 Phase classification

Basal body temperature was measured every morning upon waking up with a gynecological thermometer for approximately one month from the first day of menstruation to the beginning of the following month. An obstetrician and several midwives estimated ovulation periods based on the subjects’ temperatures (**Figure S1**). For some of participants, we also measured salivary progesterone concentrations using an ELISA kit (SALIMETRICS) to estimate periods as complementary data (**Figure S2**). Because the periods of Subject #F2 and #F6 were not estimated, their data were excluded. The luteal phase was divided into two subphases because the first and second halves of a luteal phase can be affected by the follicular cycle and the upcoming menstruation, respectively. In addition, the menstrual cycle was divided into four phases: menstrual, follicular, early luteal, and late luteal phase (**Figure S1**). Male subjects used samples from the periods corresponding to the number of days (first duration, Days 1–2; second duration, Day 14; third duration, Day 21–22; fourth duration, Day 28).

### 2.4 Next generation sequencing and microbiome analysis

We prepared libraries of the V3–V4 region of the 16S rRNA gene with KAPA HiFi HS ReadyMix (KAPA BIOSYSTEMS) using the salivary DNAs as templates according to the 16S rRNA metagenomics protocol for MiSeq system (Illumina). The PCR samples were cleaned up by AMPure XP beads (Beckman Coulter) and the read data were obtained by a MiSeq sequencer using Miseq Reagent Kit v3 and PhiX Control v3 (Illumina). The raw reads data have been registered with DNA Data Bank of Japan under the accession number DRA015154 (**Table S1**). The obtained read data were analyzed by the QIIME2 program (Caporaso et al., 2010) (https://qiime2.org/). After merging the pair end reads, quality check and chimera checks were conducted with the DADA2 plugin (Callahan et al., 2016) . To analyze taxonomy, a classifier was trained using SILVA 16S rRNA database (Ver. 123) (Quast et al., 2013). The samples were rarefied at 10,000 depths of sequences.

### 2.5 Quantitative PCR

Quantitative PCR targeting part of the 16S rRNA gene (regions 321–524) was performed using Quantifast SYBR kit (QIAGEN) with a pair of primers: Forward 5’-(ACTGAGAYACGGYCCA)-3’ and Reverse 5’-(CTGCTGGCACGDAGTTAGCC)-3’ (Wang and Qian, 2009). The copy numbers of the gene in saliva were calculated using the indicated copy numbers of the *Staphylococcus caprae* strain JMUB145, which contains six copies of 16S rRNA genes in a chromosome (Watanabe et al., 2018). The bacterial amount was calculated by multiplying the relative abundance ratio by the copy number.

### 2.6 Statistical analysis

Data were expressed as box plots (the middle horizontal line refers to the median value and the width of the box represents the interquartile range), and all statistical analyses were performed in SPSS version 24 (IBM) or R version 4.1.1. Alpha diversity was calculated as the observed number of species as well as Simpson, Shannon, and Chao1. Differences among menstrual cycles or trimesters were calculated with the Friedman test, followed by *post hoc* pairwise comparisons using the Bonferroni test. Correlations were calculated with Spearman’s rank correlation coefficient test. Beta diversity was calculated on Bray-Curtis distances and clustered on complete linkage. The factors that affect bacteria compositions on beta diversity assessed through a permutational multivariate analysis (PERMANOVA). A p-value < 0.05 was considered significant.

## 3 Results

### 3.1 Demographic data of subjects

The saliva samples were taken from healthy young adult females (aged: 23–36 years, body mass index: 18.0–30.6, and menstrual duration: 25–43 days), and males (aged: 22–32 years and body mass index: 18.7–34.5). All subjects had no oral problems, and no habit of using dental floss (**Table S1**).

### 3.2 Genera abundances in the oral microbiome

**Figure 1:**
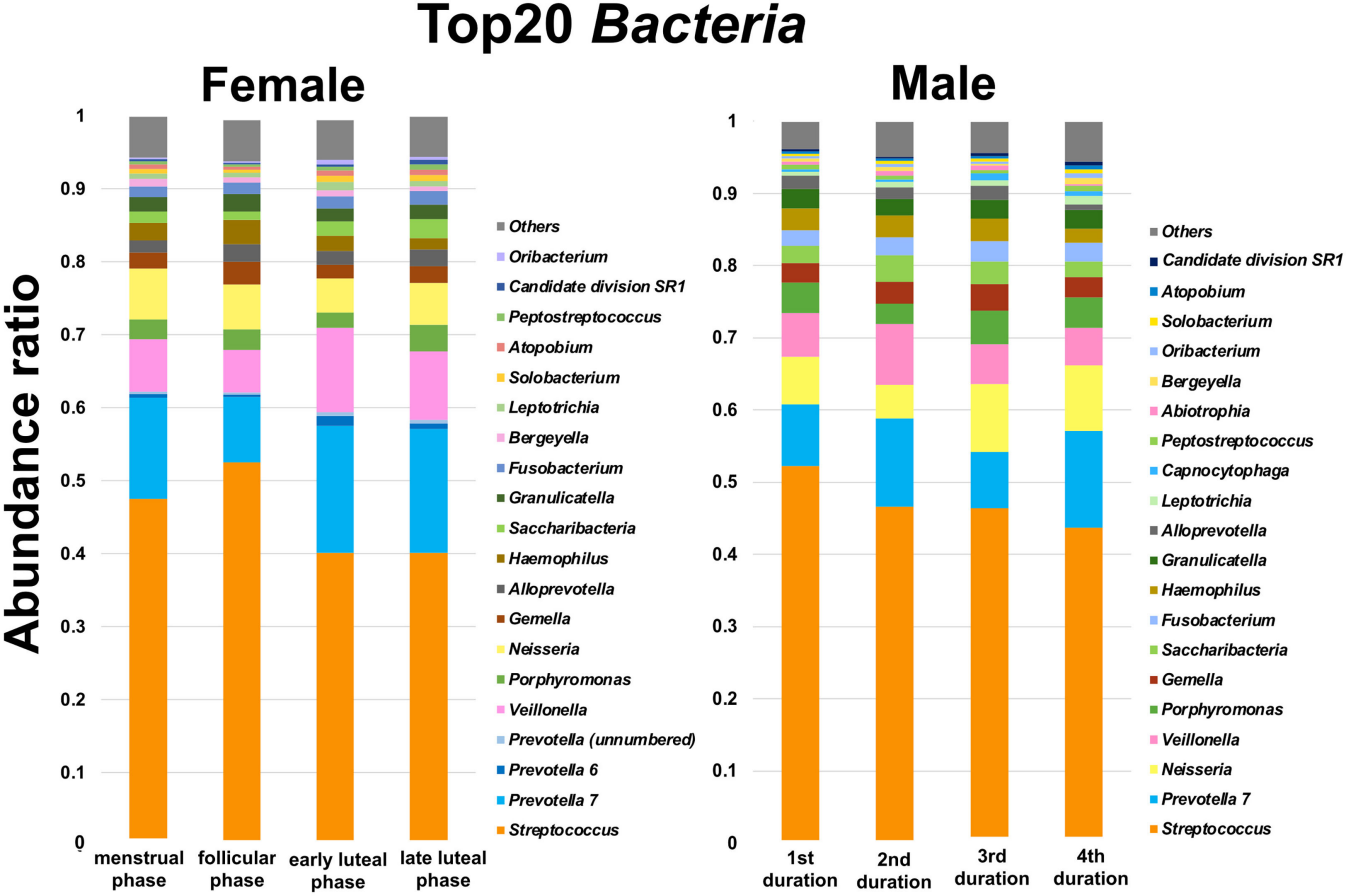
Abundance ratios of the top 20 bacteria genera in the oral microbiome. Stacked bar plots showing the composition of the most dominant genera color-coded and ranked according to abundance, while *Prevotella 6* and Prevotella unnumbered genera were arranged next to *Prevotella 7* genus. The abundance ratios in females are shown in four phases: menstrual, follicular, early luteal, and late luteal phases. The abundance ratios in males are shown with four durations corresponding to the number of days: days 1–2, 1^st^ duration; day 14, 2^nd^ duration; days 21–22, 3^rd^ duration; day 28, 4^th^ duration. Females, N = 9; Males, N = 5.

**Figure 2:**
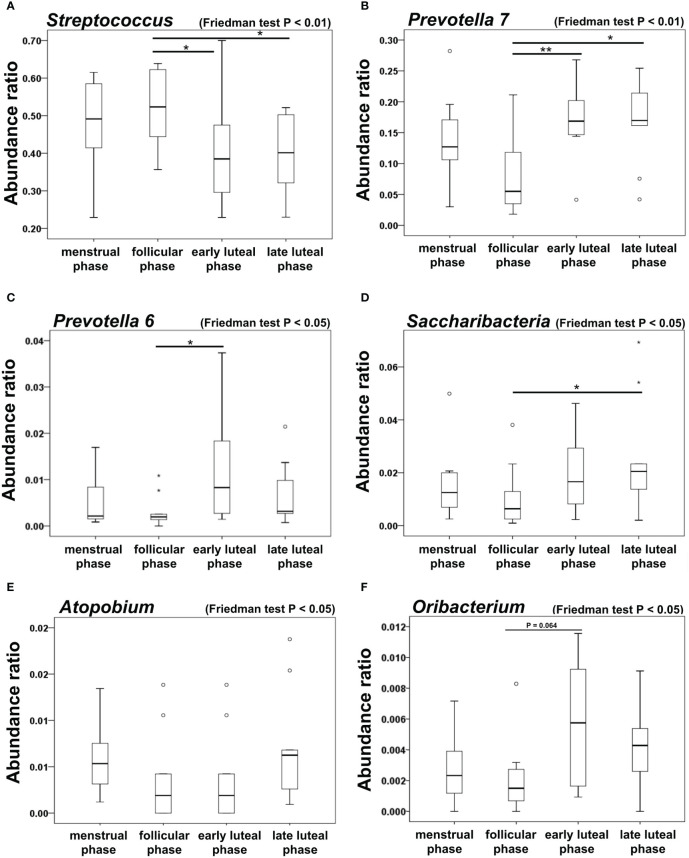
Abundance ratios of each bacterial genus in the oral microbiome of female subjects. Boxplots showing the abundance ratios of each bacterial genus during menstrual cycles: **(A)** *Streptococcus* genera. **(B)** *Prevotella 7* genera. **(C)** *Prevotella 6* genera, **(D)** *Saccharibacteria* genera, **(E)** *Atopobium* genera, and **(F)** *Oribacterium* genera (menstrual, follicular, early luteal, and late luteal phases). Friedman test and Bonferroni test, ^*^ *P <*0.05 and ^**^ *P* < 0.01. N = 9.

The oral microbiome in both females and males mainly consists of *Streptococcus*, *Prevotella*, *Veillonella*, *Porphorymonas*, *Nesseria*, and *Gemella* genera (**Figure 1**). With respect to the *Prevotellaceae* family, the *Prevotella* 7, *Prevotella* 6, and *Prevotella* (unnumbered) genera were detected. Species in the *Prevotella* 7, *Prevotella* 6, and *Prevotella* (unnumbered) genera in the SILIVA database (Ver. 123) are listed in **Table S2**. Among the abundance ratios of the top 20 oral microbiomes, Friedman tests revealed significant differences in five genera among females. The abundance ratio of the *Streptococcus* genus in the follicular phase was significantly higher than that in the early and late luteal phases (*P* < 0.05, **Figure 2A**), while the abundance ratios of the *Prevotella* 7 and *Prevotella* 6 genera were significantly lower in the follicular phase than in the early and late luteal phases (*P* < 0.05, **Figure 2B**) and in the early luteal phase (*P* < 0.05, **Figure 2C**), respectively. The abundance ratio of the *Saccharibacteria* genus was significantly lower in the follicular phase than in the late luteal phase (*P* < 0.05, **Figure 2D**). While Friedman test detected significant difference in the abundance ratio of Atopobium genus, there was no significant difference by post hoc pariwise comparisons (**Figure 2E**). Furthermore, the abundance ratio of the Oribacteirum genera tended to be lower in the follicular phase than in the early lutealphase (*P* = 0.064, **Figure 2F**). In contrast, there were no significant differences in the abundance ratios of genera among males.

### 3.3 Alpha diversity and beta diversity

**Figure 3:**
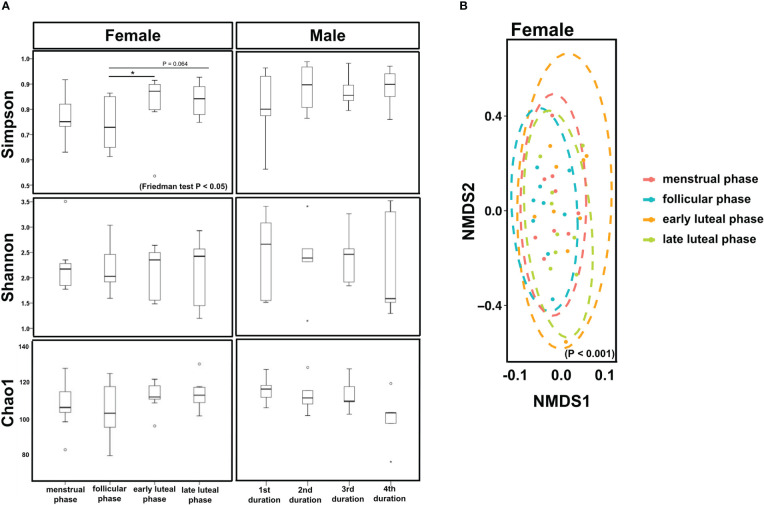
Bacterial diversity. **(A)** Box plots showing the alpha diversity indices, Simpson, Shannon, and Chao1, based on the phases of the menstrual cycles (menstrual, follicular, early luteal, and late luteal phases) or durations (days 1–2, 1^st^ duration; day 14, 2^nd^ duration; days 21–22, 3^rd^ duration; day 28, 4^th^ duration). Friedman test and Bonferroni test, ^*^ *P <*0.05. Females, N = 9; Males, N = 5. **(B)** Nonmetric multidimensional scaling plot showing the dissimilarities of all samples collected in this study according to menstrual cycles (menstrual, follicular, early luteal, and late luteal phases). Ellipsoids represent a 95% confidence interval used to group each sample.

In females, the Simpson index was significantly lower in the follicular phase than in the early luteal phase (*P* < 0.05, **Figure 3A**) and tended to be lower than in the late luteal phase (*P* = 0.064, **Figure 3A**), while neither the Shannon nor the Chao1 index showed significant differences among the four phases. In contrast, beta diversity revealed significant differences among the four phases (*P* < 0.01, **Figure 3B**). There were no significant differences among the periods in males.

### 3.4 Copy numbers of the 16S rRNA genes

**Figure 4:**
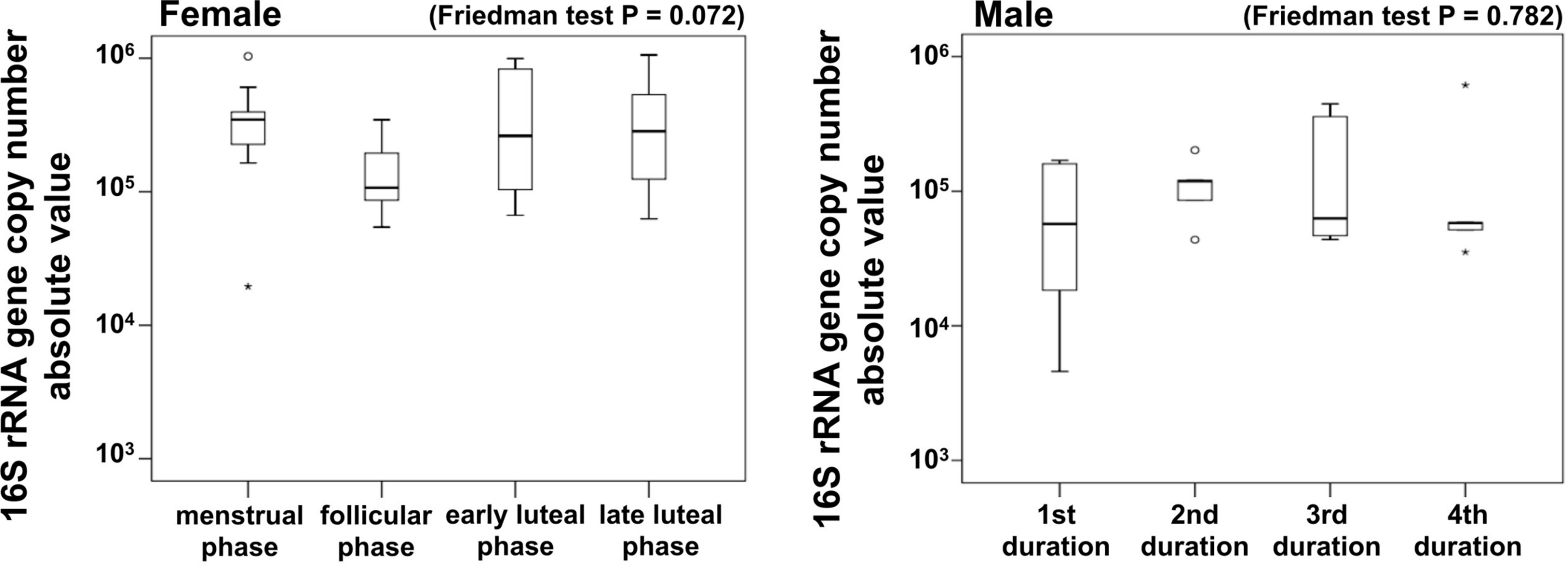
Copy numbers of the 16S rRNA gene collected by saliva samples. Data is shown in box plots. Friedman test and Bonferroni test. Females, N = 9; Males, N = 5. The vertical axis was a base-10 log scale. rRNA, ribosomal RNA.

In order to examine changes in bacterial amounts during the menstrual cycles, the copy numbers of the 16S rRNA genes were calculated by quantitative PCR (**Figure 4**). In females, Friedman test revealed that the 16S rRNA gene copy number absolute value tended to be difference (*P* = 0.072, **Figure 4**). In contrast, there were no significant differences in the copy numbers of the 16 S rRNA gens among males.

### 3.5 Bacterial amounts

**Figure 5:**
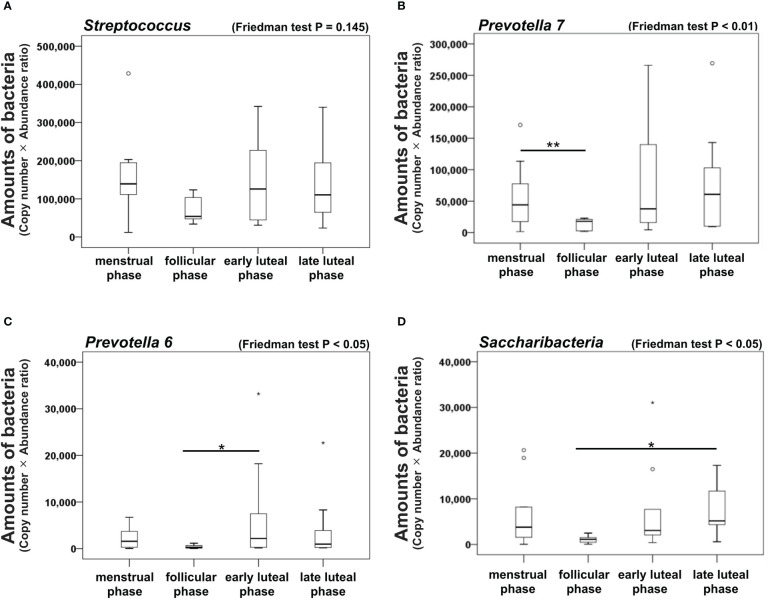
Amounts of dominant bacteria genera in the oral microbiome of female subjects. Box plots showing the amounts of **(A)** *Streptococcus*, **(B)** *Prevotella 7* genera, **(C)** *Prevotella 6*, and **(D)** *Saccharibacteria* genera during the menstrual cycles. Friedman test and Bonferroni test, *P < 0.05 and ^**^ *P* < 0.01. N = 9.

To estimate changes in the numbers of each bacterial genus during the menstrual cycles, we multiplied the copy numbers by the abundance ratio. Our results demonstrate there were no significant differences in the amount of the *Streptococcus* genus among the four phases (**Figure 5A**). In contrast, the amount of the *Prevotella* 7 genus was significantly lower in the follicular phase than in the menstrual phase (*P* < 0.01, **Figure 5B**) while that of the *Prevotella 6* genus was significantly lower in the follicular phase than in the early luteal phase (*P* < 0.05, **Figure 5C**) but not in the late luteal phase. Finally, the total amount of the *Saccharibacteria* genus was significantly lower in the follicular phase than in the late luteal phase (*P* < 0.05, **Figure 5D**).

### 3.6 Correlation of bacteria genera in the oral microbiome

The Spearman’s rank correlation coefficients were r = −0.850, −0.783, and −0.850 in the menstrual, follicular, and early luteal phase, respectively, indicating a negative correlation between the *Streptococcus* and *Prevotella 7* genera (*P* < 0.05), while there was no significant correlation in the late luteal phase (**Table 1**). Correlation between the *Streptococcus* genus and *Prevotella 6* genera was observed only in the late luteal phase (r = −0.717, *P* < 0.05). There was no correlation between the *Streptococcus* genus and *Prevotella* (unnumbered) genera in the four phases. Although *Prevotella (unnumbered)* and *Porphyromonas* genera tended to be positively correlated in the early luteal phase (r = 0.6, *P* = 0.088), the abundance ratios were correlated negatively in the follicular phase (r = −0.817, *P* < 0.05) (**Table S3**).

**Table 1:** Correlation of the abundance ratios of bacterial genera in menstrual cycles.

	Menstrual	Follicular	Early Luteal	Late Luteal
Streptococcus/Prevotella 7	−0.850^*^	−0.783^*^	−0.850^**^	−0.433
Streptococcus/Prevotella 6	−0.433	−0.583	−0.100	−0.717^*^
Streptococcus/Prevotella (unnumbered)	−0.533	−0.150	−0.483	−0.417

Spearman’s rank correlation coefficient test, ^*^P < 0.05 and ^**^P < 0.01. N = 9.

## 4 Discussion

Several studies have addressed the changes in the oral environment during the menstrual cycle (Baser et al., 2009; Balan et al., 2012; Khosravisamani et al., 2014). However, the relationship between the menstrual cycle and the oral microbiome has not been clarified. Therefore, in this study, we used 16S rRNA-based sequencing to examine the cyclical changes in oral microbiome and assess their impact on females’ health and wellbeing. Our study revealed the presence of various microorganisms during the menstrual cycles of healthy young adult females, indicating reciprocal changes with the *Streptococcus* genus and *Prevotella* genera, especially in the follicular phase. To the best of our knowledge, this is the first study to prove the cyclical changes with specific bacteria genus during the menstrual cycle by 16S rRNA-based sequencing. The results of this study may provide a basis for etiological studies of female hormone-influenced oral disorders.

The main finding of this study is that changes in the *Streptococcus* and *Prevotella* genera abundances in healthy young adult females in Japan occur in accordance with their menstrual cycles. Previously, Bostanci et al. reported that the abundances of these bacteria were not altered during the menstrual cycles in adult females in Denmark (Bostanci et al., 2021). This discrepancy might be attributed to the methods used for the collection of samples. In this study, we collected saliva samples immediately after waking, while Bostanci et al. collected their samples after 30 minutes of fasting in the hospital. In addition, dietary and racial differences could also be associated with this discrepancy. In our study, bacteria of the *Streptococcus* genus were the most predominant followed by bacteria of the *Prevotella 7* genus. In a previous report, bacteria of the *Prevotella* genus (including all *Prevotella 1—9* and unnumbered genera) were the most abundant followed by bacteria of the *Streptococcus* genus, indicating that the effect of menstrual cycle correlates with the composition of the oral microbiome.

Alpha diversity represents microbiome richness and community diversity in a sample or site, and it is calculated by different formulas based on the Simpson, Shannon, and Chao1 indices. Generally, the Simpson index gives more weight to higher abundant genera. In our study, compared with the Shannon or Chao1 index, the Simpson index revealed was significantly decreased in the follicular phase, suggesting notable changes in the predominant *Streptococcus* and *Prevotella* genera. To our knowledge, there are no variations in the alpha diversity of the oral microbiome between healthy young people and depression. However, it has been previously reported that the Shannon index is different in the depressed group than in healthy adults, although there was no significant difference in the mean values of the diversity (Wingfield et al., 2021). Several reports concluded that there was no significant difference in the alpha diversity of oral microbiome between pregnant and nonpregnant groups (Sparvoli et al., 2020), between patients with anxiety and depression and control subjects *(* Simpson et al., 2020), or between postpartum and nonpregnant females (Khadija et al., 2021). Further analysis is required to clarify whether changes in the alpha diversity of oral microbiome depend on the oral environment, disease, and/or mouth conditions.

Furthermore, the present study found that the amount of the *Prevotella 7* genus significantly decreased in the follicular phase. During that phase, estradiol reaches its highest levels, as opposed to progesterone, which reaches its highest levels during the early luteal phase. Previous studies have investigated the relationships between female hormones (estradiol and progesterone) and bacterial amounts. For example, ovariectomy in mice showed that oral bacterial amounts increased as a result of estradiol deficiency (Lucisano et al., 2021), although this report did not focus on *Prevotella* species. In addition, *Prevotella* species have been found to change their characteristics according to estradiol levels (Könönen et al., 2022), of which *Prevotella intermedia*, *Prevotella nigrescens*, and *Prevotella pallens* increased biofilm formation in the presence of estradiol (Fteita et al., 2015; Fteita et al., 2017). The decrease observed in the *Prevotella 7* genus in saliva might be due to the fact that estradiol-induced biofilm prevents *Prevotella* from flowing into the saliva. Regarding progesterone, *Prevotella* species as well as *Porphyromonas gingivalis* utilize progesterone as a source of nutrition (Basavaraju et al., 2012; Butera et al., 2021), indicating that the increase in the amount of *Prevotella 7* genus in the late luteal phase is due to a progesterone-dependent growth. Further studies are required to identify the mechanisms with which female hormones influence the amount of *Prevotella* species.

Interestingly, the *Prevotella 7* genus was negatively correlated with the *Streptococcus* genus in the menstrual, follicular, and early luteal phases, and the *Prevotella* 6 genus was negatively correlated with the *Streptococcus* genus in the late luteal phase. These results demonstrate that the *Prevotella* genera are associated with the menstrual cycle and/or other bacterial genera under different regulations. According to the SILIVA database (Ver. 123), *Prevotella 7* contains *P. buccae*, *P. dentasini*, *P. denticola*, *P. enoeca*, and *P. melaninogenica* (**Table S2**). Among them, *P. melaninogenica* was found in high proportions in the saliva of 225 systemically healthy individuals (Mager et al., 2003), although this species is recognized as pathogenic. However, it remains unknown whether the increase and decrease in *P. melaninogenica* and other *Prevotella 7* genera in this study affected the health status of the females. *P. intermedia* and *P. nigrescens*, members of the *Prevotella (unnumbered)* (**Table S2**), are associated with inflammatory periodontal diseases together with *Porphyromonas gingivalis* (Lafaurie et al., 2017; Iuşan et al., 2022). Although the abundance ratio of *Prevotella (unnumbered)* and *Porphyromonas* tended to be positively correlated in the early luteal phase, the abundance ratios were significantly negative in the follicular phase (**Table S3**). These results indicate that the menstrual cycle is associated with periodontal diseases through affecting interaction between *Prevotella (unnumbered)* and *Porphyromonas*.

The SILVA database is a comprehensive web resource for up-to-date, quality-controlled databases of aligned rRNA gene sequences from the Bacteria, Archaea and Eukaryota domains (Quast et al., 2013). Several previous studies have also used the SILVA database for oral microbiome analysis (Kennedy et al., 2016; Zhao et al., 2021; Zhao et al., 2022). Furthermore, a recent systematic review of oral peri-implant and periodontal microbiota (Gazil et al., 2022) showed a specific database for oral microbiomes, such as the HOMD (Chen et al., 2010) (https://www.homd.org/). Further analysis using oral-specific databases, such as the HOMD would be interesting.

### Limitations

Our study has several limitations. First, only saliva, but not intragingival, samples were collected. Consequently, it is unclear whether our results do reflect the bacterial flora in the gingiva. Second, the subjects included in this study had no oral issues; therefore, it is unclear how the affected oral microbiome in subjects with oral problems may influence our results. Third, our sample size was small and limited (only included Japanese subjects), restricting the generalization of our present findings. Finally, our participants included one female with overweight and one male with obesity, thereby restricting the generalization of our present findings.

## 5 Conclusion

Oral microbiome profiling in menstrual cycles using 16S rRNA sequencing revealed the presence of a community of microorganisms in healthy young adult females, indicating reciprocal changes with the *Streptococcus* and *Prevotella* genera, especially in the follicular phase. Further studies are required to identify the mechanism with which these cyclical changes affect oral microbiome.

## Data availability statement

The data presented in the study are deposited in the repository of DNA Data Bank of Japan (DDBJ), accession number DRA015154.

## Ethics statement

The studies involving human participants were reviewed and approved by Medical Ethics Committee of Kanazawa University (Approval No. 953-1). The patients/participants provided their written informed consent to participate in this study. This study was carried out in accordance with the Ethical Principles for Medical Research Involving Human Subjects (Declaration of Helsinki), the Ethical Guidelines for Medical Research Involving Human Subjects (Ministry of Health, Labor and Welfare), the Regulations on Medical Research Involving Human Subjects at Kanazawa University, and the Kanazawa University Regulations for Prevention of Misconduct in Research Activities.

## Author contributions

AY conducted the ELISA experiments and protein concentration calculation, analyzed all data, and wrote this manuscript. KOgu analyzed the sequence data and revised the draft of this manuscript. KMi analyzed body temperatures and the ELISA data to determine the menstrual cycles. KOga performed DNA extraction and sequencing processes. TH collected the saliva samples and clinical data. SO supervised AY and contributed to the discussion part. KMu conceived the study, collected the saliva samples and clinical data, conducted part of the experiments, and revised the manuscript critically for content. All authors reviewed and accepted the manuscript.

## References

[B1] Balan U. Gonsalves N. Jose M. Girish K.L. (2012). Symptomatic changes of oral mucosa during normal hormonal turnover in healthy young menstruating female. J. Contemp. Dent. Pract. 13, 178–181. doi: 10.5005/jp-journals-10024-1117 22665744

[B2] Basavaraju A. Durga S. V. Vanitha B. (2012). Variations in the oral anaerobic microbial flora in relation to pregnancy. J. Clin. Diagn. Res. 6, 1489–1491. doi: 10.7860/JCDR/2012/4609.2540 23285437 PMC3527777

[B3] Baser U. Cekici A. Tanrikulu-Kucuk S. Kantarci A. Ademoglu E. Yalcin F. (2009). Gingival inflammation and interleukin-1 beta and tumor necrosis factor-alpha levels in gingival crevicular fluid during the menstrual cycle. J. Periodontol. 80, 1983–1990. doi: 10.1902/JOP.2009.090076 19961381

[B4] Borgo P. V. Rodrigues V. A. A. Feitosa A. C. R. Xavier K. C. B. Aliva-Campos M. J. (2014). Association between periodontal condition and subgingival microbiota in female during pregnancy: a longitudinal study. J. Appl. Oral. Sci. 22, 528–533. doi: 10.1590/1678-775720140164 25591021 PMC4307767

[B5] Bostanci N. Krog M. C. Hugerth L. W. Bashir Z. Fransson E. Boulund F.. (2021). Dysbiosis of the human oral microbiome during the menstrual cycle and vulnerability to the external exposures of smoking and dietary sugar. Front. Cell. Infect. Microbiol. 11. doi: 10.3389/FCIMB.2021.625229 PMC8018275 33816334

[B6] Butera A. Maiorani C. Morandini A. Simonini M. Colnaghi A. Morittu S.. (2021). Assessment of oral microbiome changes in healthy and COVID-19-Affected pregnant female: A narrative review. Microorganisms 9, 2385. doi: 10.3390/microorganisms9112385 PMC8618476 34835510

[B7] Calil C. M. Lima P. O. Bernardes C. F. Groppo F. C. Bado F. Marcondes F. K. (2008). Influence of gender and menstrual cycle on volatile sulphur compounds production. Arch. Oral. Biol. 53, 1107–1112. doi: 10.1016/J.archoralbio.2008.06.008 18691697

[B8] Callahan B. J. McMurdie P. J. Rosen M. J. Han A. W. Johnson A. J. A. Holmes S. P. (2016). DADA2: High-resolution sample inference from illumina amplicon data. Nat. Methods 13 (7), 581–583. doi: 10.1038/nmeth.3869 27214047 PMC4927377

[B9] Caporaso J. G. Kuczynski J. Stombaugh J. Bittinger K. Bushman F. D. Costello E. K.. (2010). QIIME allows analysis of high-throughput community sequencing data. Nat. Methods 7 (5), 335–336. doi: 10.1038/nmeth.f.303 20383131 PMC3156573

[B10] Chen T. Yu W. H. Izard J. Baranova O. V. Lakshmanan A. Dewhirst F. E. (2010). The human oral microbiome database: a web accessible resource for investigating oral microbe taxonomic and genomic information. Database (Oxford), 2010, baq013. doi: 10.1093/database/baq013 20624719 PMC2911848

[B11] Dewhirst F. E. Chen T. Izard J. Paster B. J. Tanner A. C. R. R. Yu W. H.. (2010). The human oral microbiome. J. Bacteriol. 192, 5002–5017. doi: 10.1128/JB.00542-10 20656903 PMC2944498

[B12] Farage M. A. Osborn T. W. MacLean A. B. (2008). Cognitive, sensory, and emotional changes associated with the menstrual cycle: A review. Arch. Gynecol. Obstet. 278, 299–307. doi: 10.1007/S00404-008-0708-2 18592262

[B13] Fischer C. C. Persson R. E. Persson G. R. (2008). Influence of the menstrual cycle on the oral microbial flora in female: A case-control study including men as control subjects. J. Periodontol. 79, 1966–1973. doi: 10.1902/JOP.2008.080057 18834253

[B14] Fteita D. Könönen E. Gürsoy M. Söderling E. Gürsoy U. K. (2015). Does estradiol have an impact on the dipeptidyl peptidase IV enzyme activity of the prevotella intermedia group bacteria? Anaerobe 36, 14–18. doi: 10.1016/j.anaerobe.2015.09.002 26386229

[B15] Fteita D. Musrati A. A. Könönen E. Ma X. Gürsoy M. Peurla M.. (2017). Dipeptidyl peptidase IV and quorum sensing signaling in biofilm-related virulence of prevotella aurantiaca. Anaerobe 48, 152–159. doi: 10.1016/j.anaerobe.2017.08.009 28821458

[B16] Fujiwara N. Tsuruda K. Iwamoto Y. Kato F. Odaki T. Yamane N.. (2017). Significant increase of oral bacteria in the early pregnancy period in Japanese female. J. Investig. Clin. Dent. 8, e12189. doi: 10.1111/JICD.12189 26345599

[B17] Gao L. Xu T. Huang G. Jiang S. Gu Y. Chen F. (2018). Oral microbiomes: more and more importance in oral cavity and whole body. Protein Cell 9, 488. doi: 10.1007/S13238-018-0548-1 29736705 PMC5960472

[B18] Gazil V. Bandiaky O. N. Renard E. Idiri K. Struillou X. Soueidan A. (2022). Current data on oral peri-implant and periodontal microbiota and its pathological changes: A systematic review. Microorganisms 10 (12), 2466. doi: 10.3390/microorganisms10122466 36557719 PMC9781768

[B19] Iuşan S. A. L. Lucaciu O. P. Petrescu N. B. Mirică I. C. Toc D. A. Albu S.. (2022). The main bacterial communities identified in the sites affected by periimplantitis: A systematic review. Microorganisms 10, 1232. doi: 10.3390/microorganisms10061232 PMC9228476 35744750

[B20] Kawamoto A. Sugano N. Motohashi M. Matsumoto S. Ito K. (2010). Relationship between oral malodor and the menstrual cycle. J. Periodontal Res. 45, 681–687. doi: 10.1111/J.1600-0765.2010.01285.x 20572919

[B21] Kawamoto A. Sugano N. Motohashi M. Matsumoto S. Ito K. (2012). Relationship between salivary antioxidant capacity and phases of the menstrual cycle. J. Periodontal Res. 47, 593–598. doi: 10.1111/J.1600-0765.2012.01471.x 22376058

[B22] Kennedy R. Lappin D. F. Dixon P. M. Buijs M. J. Zaura E. Crielaard W.. (2016). The microbiome associated with equine periodontitis and oral health. Vet. Res. 47, 49. doi: 10.1186/s13567-016-0333-1 27080859 PMC4832512

[B23] Khadija B. Badshah L. Siddiqa A. Rehman B. Anjum S. Saeed A.. (2021). Dysbiosis in salivary bacterial diversity of postpartum females and its association with oral health problems and APOs. Curr. Res. Microb. Sci. 2, 100032. doi: 10.1016/j.crmicr.2021.100032 34841323 PMC8610344

[B24] Khosravisamani M. Maliji G. Seyfi S. Azadmehr A. Abd Nikfarjam B. Madadi S.. (2014). Effect of the menstrual cycle on inflammatory cytokines in the periodontium. J. Periodontal Res. 49, 770–776. doi: 10.1111/jre.12161 24673464

[B25] Könönen E. Fteita D. Gursoy U. K. Gursoy M. (2022). Prevotella genus as oral residents and infectious agents with potential impact on systemic conditions. J. Oral. Microbiol. 14, 2079814. doi: 10.1080/20002297.2022.2079814 PMC9662046 36393976

[B26] Lafaurie G. I. Sabogal M. A. Castillo D. M. Rincón M. V. Gómez L. A. Lesmes Y. A.. (2017). Microbiome and microbial biofilm profiles of peri-implantitis: A systematic review. J. Periodontol. 88, 1066–1089. doi: 10.1902/jop.2017.170123 28625077

[B27] Lain K. Y. Catalano P. M. (2007). Metabolic changes in pregnancy. Clin. Obstet. Gynecol. 50, 938–948. doi: 10.1097/GRF.0b013e31815a5494 17982337

[B28] Lucisano M. P. da Silva R. A. B. de Sousa Pereira A. P. Romualdo P. C. Feres M. de Queiroz A. M.. (2021). Alteration of the oral microbiota may be a responsible factor, along with estrogen deficiency, by the development of larger periapical lesions. Clin. Oral. Investig. 25, 3651–3662. doi: 10.1007/s00784-020-03688-5 33188615

[B29] Machtei E. E. Mahler D. Sanduri H. Peled M. (2004). The effect of menstrual cycle on periodontal health. J. Periodontol. 75, 408–412. doi: 10.1902/jop.2004.75.3.408 15088879

[B30] Mager D. L. Ximenez-Fyvie L. A. Haffajee A. D. Socransky S. S. (2003). Distribution of selected bacterial genus on intraoral surfaces. J. Clin. Periodontol. 30, 644–654. doi: 10.1034/j.1600-051x.2003.00376.x 12834503

[B31] Muizzuddin N. Marenus K. D. Schnittger S. F. Sullivan M. Maes D. H. (2006). Effect of systemic hormonal cyclicity on skin. Int. J. Cosmet. Sci. 28, 77–77. doi: 10.1111/j.1467-2494.2006.00304_2.x 16258697

[B32] Pelzer E. S. Allan J. A. Theodoropoulos C. Ross T. Beagley K. W. (2012). Hormone-dependent bacterial growth, persistence and biofilm formation-a pilot study investigating human follicular fluid collected during IVF cycles. PloS One 7, 49965. doi: 10.1371/journal.pone.0049965 PMC3514270 23226503

[B33] Quast Q. Pruesse E. Yilmaz P. Gerken J. Schweer T. Yarza P.. (2013). The SILVA ribosomal RNA gene database project: improved data processing and web-based tools. Nucleic Acids Res. 41, D590–D596. doi: 10.1093/nar/gks1219 23193283 PMC3531112

[B34] Saadaoui M. Singh P. Al Khodor S. (2021). Oral microbiome and pregnancy: A bidirectional relationship. J. Reprod. Immunol. 145, 103293. doi: 10.1016/j.jri.2021.103293 33676065

[B35] Simpson C. A. Adler C. du Plessis M. R. Landau E. R. Dashper S. G. Reynolds E. C.. (2020). Oral microbiome composition, but not diversity, is associated with adolescent anxiety and depression symptoms. Physiol. Behav. 226, 113126. doi: 10.1016/j.physbeh.2020.113126 32777312

[B36] Sparvoli L. G. Cortez R. V. Daher S. Padilha M. Sun S. Y. Nakamura M. U.. (2020). Female’s multisite microbial modulation during pregnancy. Microb. Pathog. 147, 104230. doi: 10.1016/j.micpath.2020.104230 32428665

[B37] Verma D. Garg P. K. Dubey A. K. (2018). Insights into the human oral microbiome. Arch. Microbiol. 2018 2004 200, 525–540. doi: 10.1007/s00203-018-1505-3 29572583

[B38] Wang Y. Qian P. Y. (2009). Conservative fragments in bacterial 16S rRNA genes and primer design for 16S ribosomal DNA amplicons in metagenomic studies. PloS One 4 (10), e7401. doi: 10.1371/journal.pone.0007401 19816594 PMC2754607

[B39] Wang Q. Würtz P. Auro K. Mäkinen V. P. Kangas A. J. Soininen P.. (2016). Metabolic profiling of pregnancy: Cross-sectional and longitudinal evidence. BMC Med. 14, 1–14. doi: 10.1186/s12916-016-0733-0 27955712 PMC5153817

[B40] Watanabe M. Kamae Y. Shiogama H. DeAngelis A. Suzuki K. (2018). Low clouds link equilibrium climate sensitivity to hydrological sensitivity. Nat. Climate Change. 8, 901–906. doi: 10.1038/s41558-018-0272-0

[B41] Wingfield B. Lapsley C. McDowell A. Miliotis G. McLafferty M. O’Neill S. M.. (2021). Variations in the oral microbiome are associated with depression in young adults. Sci. Rep. 11, 15009. doi: 10.1038/s41598-021-94498-6 PMC8298414 34294835

[B42] Zhao Y. Feng Y. Ye Q. Hu J. Feng Y. Ouyang Z.. (2022). The oral microbiome in young women at different stages of periodontitis: Prevotella dominant in stage III periodontitis. Front. Cell Infect. Microbiol. 12. doi: 10.3389/fcimb.2022.1047607 PMC9753221 36530443

[B43] Zhao J. Zhou Y. H. Zhao Y. Q. Feng Y. Yan F. Gao Z. R.. (2021). Gender variations in the oral microbiomes of elderly patients with initial periodontitis. J. Immunol. Res. 2021, 7403042. doi: 10.1155/2021/7403042 34859107 PMC8632398

