# Supplementary material for: Oral microbiome changes associated with the menstrual cycle in healthy young adult females
Source: Front Cell Infect Microbiol. 2023 Mar 31;13:1119602. doi: 10.3389/fcimb.2023.1119602 (PMC10102642; doi:10.3389/fcimb.2023.1119602)
Supplement: Supplementary file 4 [file DataSheet_1.pdf]

Supplementary Material

Oral Microbiome Changes Associated with the Menstrual Cycle in Healthy Young Adult Females

Ayaka Yamazaki[1], Kohei Ogura[2], Kana Minami[3], Kazuhiro Ogai[4], Tomomi Horiguchi[5], Shigefumi Okamoto[2,6], Kanae Mukai[5*]

*** Correspondence:** kanae_m@staff.kanazawa-u.ac.jp

1 Supplementary Figures and Tables

1.1 Supplementary Figures

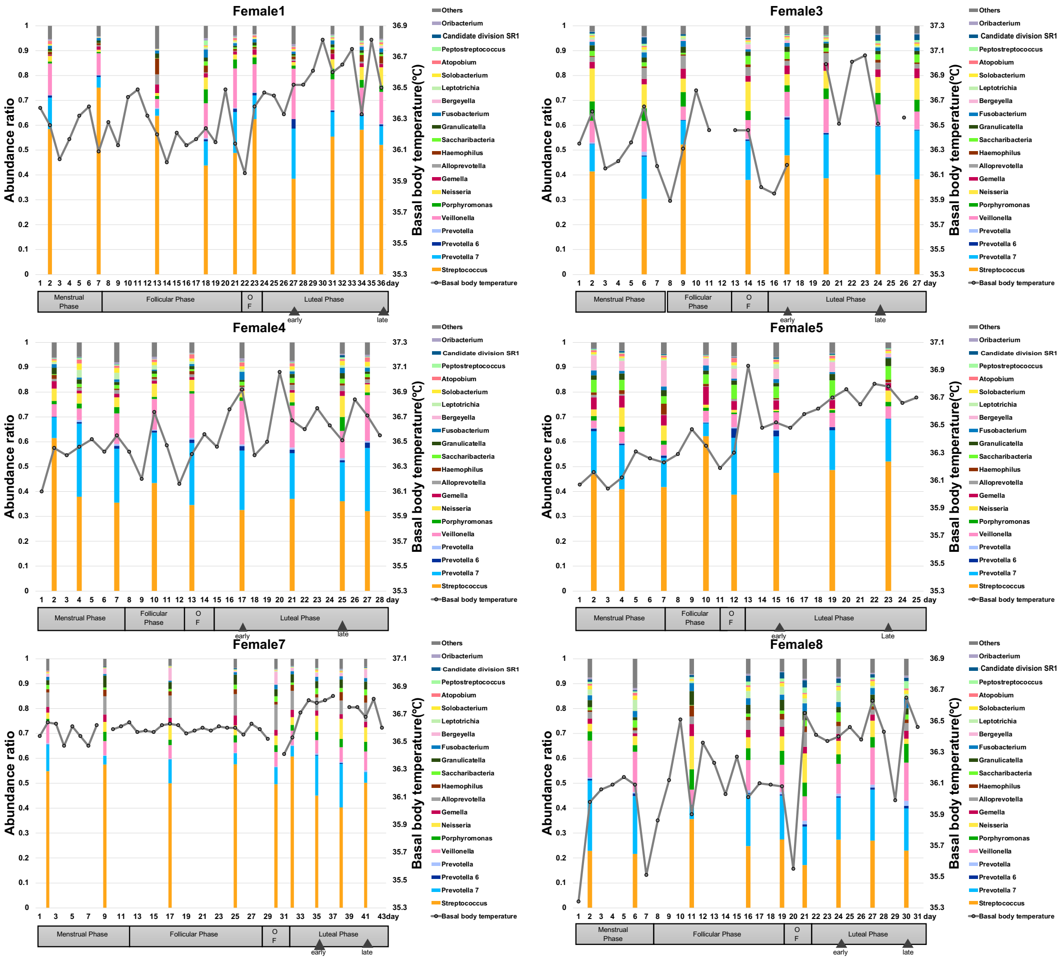

Supplementary Figure 1 (Figure S1). Phase classification and genera abundances in the oral microbiome by individuals in females. Stacked bar plots showing the composition of the most dominant genera color-coded and ranked according to abundance.

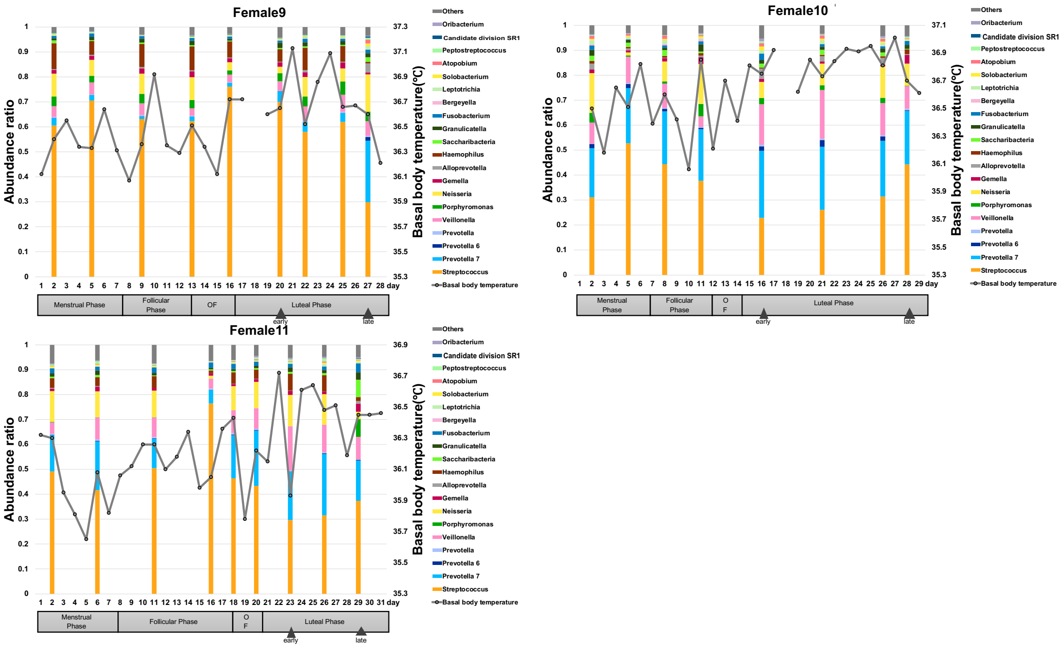

Supplementary Figure 1 continued (Figure S1). Phase classification and genera abundances in the oral microbiome by individuals in females. Stacked bar plots showing the composition of the most dominant genera color-coded and ranked according to abundance.

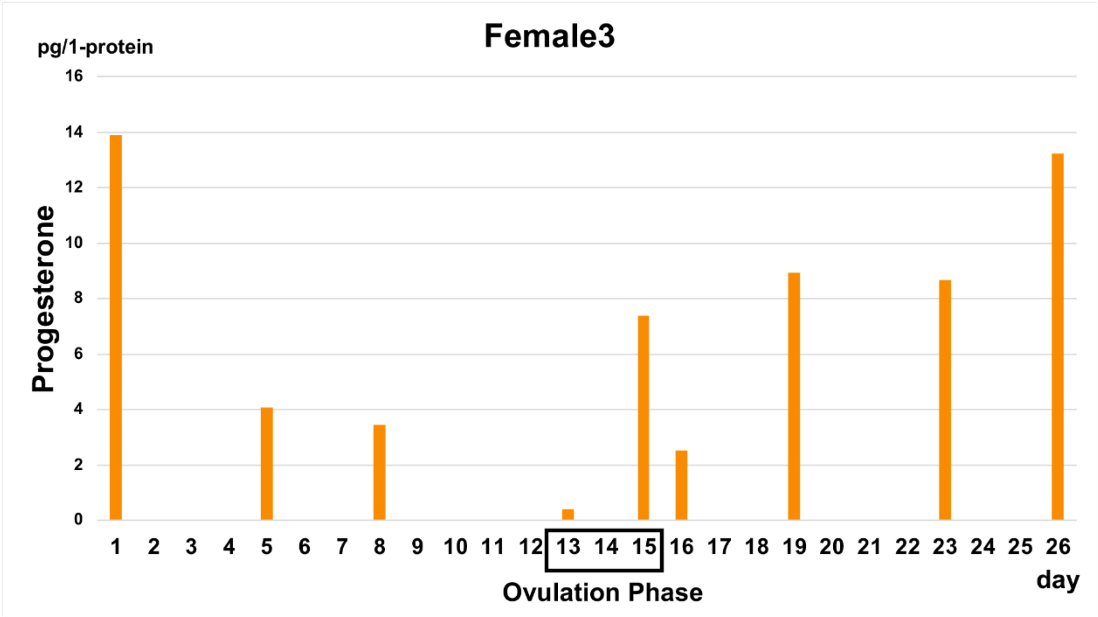

Supplementary Figure 2 (Figure S2). Salivary progesterone concentrations using an ELISA kit (SALIMETRICS) to estimate periods in female 3.

1.2 Supplementary Tables

Supplementary Table 1 (Table S1). Oral microbiome data utilized in this study

Supplementary Table 2 (Table S2). Provetella 7, Prevotella 6, and Prevotella (unnumbered) in the SILVA database (ver. 123)

Supplementary Table 3 (Table S3). Correlation of the abundance ratios of bacterial genera

frontiers
